# K-Wire-Assisted Intra-axial Intradural Approach for Cavernous Sinus Biopsy: A Case Report

**DOI:** 10.7759/cureus.74361

**Published:** 2024-11-24

**Authors:** William K Miller, Robert P Dunwoody, Jacqueline N Boyle, Andrew J Tsung

**Affiliations:** 1 Neurosurgery, University of Illinois College of Medicine Peoria, Peoria, USA

**Keywords:** biopsy, cavernous sinus, chondrosarcoma, k-wire, petroclival

## Abstract

Petroclival approaches remain challenging given abundant cranial nerves and vessels. Common trajectories include transsphenoidal, transoral, middle fossa-extradural, and posterior through the cerebellar peduncle. We report a unique intra-axial, intradural approach to the petroclival and cavernous sinus.

A 53-year-old female presented to our clinic with diplopia and a minor headache for 3 months. Imaging showed a lytic, contrast-enhancing petroclival and cavernous sinus lesion, encasing the cavernous internal carotid artery (ICA). A trajectory was planned through the superior temporal gyrus to the lateral cavernous sinus targeting the tumor margin at the cavernous sinus dural interface. A blunt attempt with the biopsy needle failed and thus a K-wire was used first to penetrate the dura followed by an exchange to the biopsy needle along the same trajectory for lesion sampling. The patient tolerated the procedure well, without any postoperative complications. Final pathology showed grade II chondrosarcoma.

To our knowledge, this case represents the first K-wire-assisted intra-axial intradural biopsy of the lateral cavernous sinus. For lesions that either do not require or are not amenable to resection due to the involvement of critical skull base structures of the cavernous region, this represents a technically straightforward cost-effective approach in tumor biopsy.

## Introduction

Clival lesions represent a challenging surgical approach for biopsy and excision given proximity to the internal carotid arteries (ICAs) and cranial nerves. Several techniques have been reported, including transsphenoidal/transoral [1,2] anteriorly, middle fossa-extradural laterally [3], and posteriorly through the cerebellar peduncle. Anterior approaches are favored for medial pathology and vice versa [4]. Both approaches risk damage to the cavernous ICA and the cranial nerves (primarily abducens) [5]. Skull base penetration, longer trajectory, and difficult angles further complicate the lateral approach [6], particularly with traditional biopsy needles.

We present a patient with a petroclival lesion with an extension into the cavernous sinus. The ICA impeded traditional petroclival approaches, thus we opted for an intra-axial approach to the cavernous sinus utilizing a Kirschner (K-wire) to penetrate the dura.

## Case presentation

**Figure 1:**
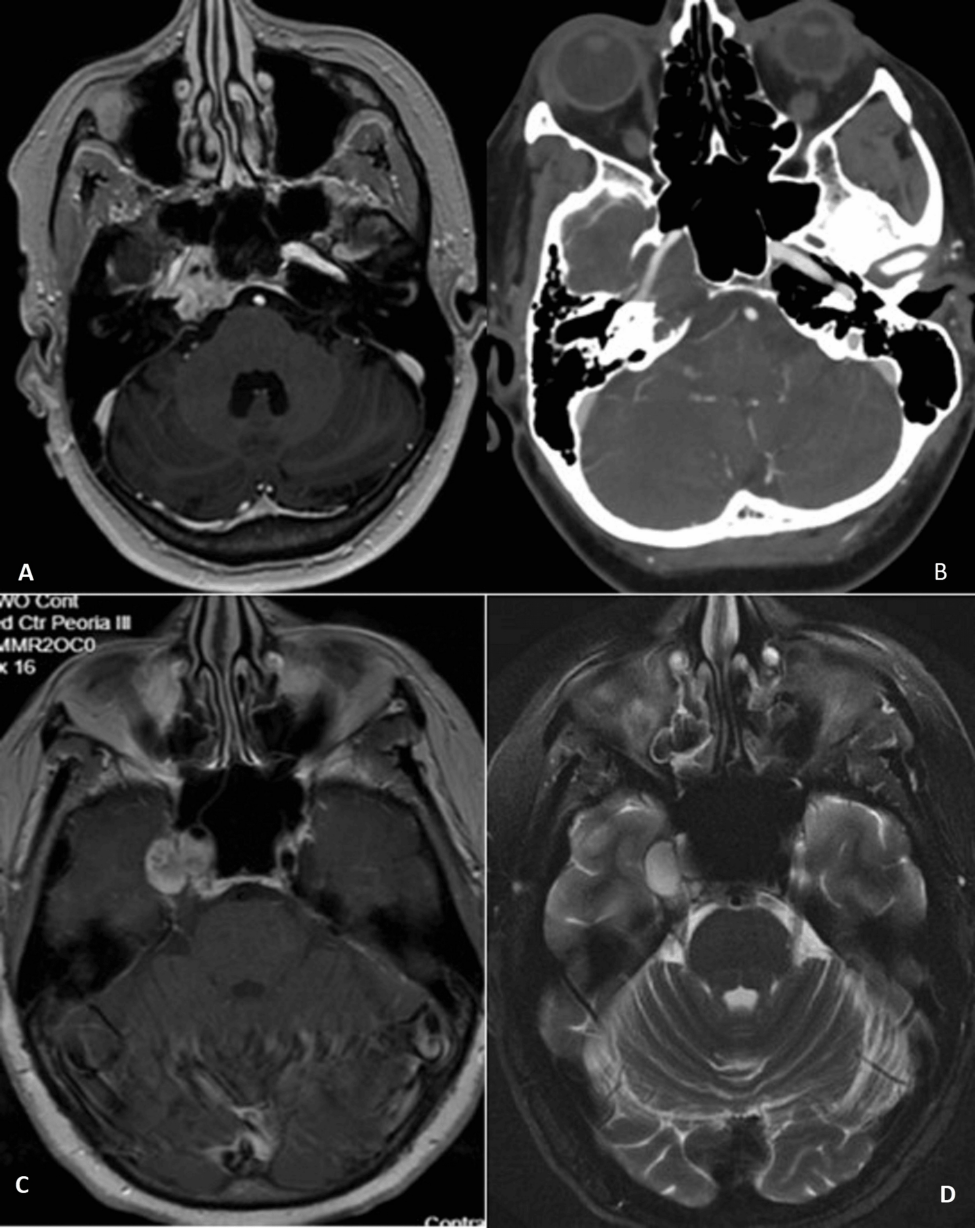
Preoperative imaging (A) T1 post-contrast sequence of the petroclival junction enhancing lesion, (B) Computed tomography angiography of the lytic lesion of the petrosal bone with overlying internal carotid artery (ICA) in the carotid canal, (C) overlying cystic component intradurally within the cavernous sinus on T1 post-contrast, and (D) T2 also showing this same cavernous sinus lesion which was targeted for biopsy.

A 53-year-old female, without pertinent past medical history, presented to our clinic with diplopia, right facial numbness, and mild headaches for approximately three months. Initial examination revealed mild right abducens palsy. CT head and MRI with and without contrast showed a lytic expansile lesion of the right petroclival junction with anterior displacement of the cavernous carotid (Figure 1A-1C). T2 sequences revealed a cystic, intradural lesion adjacent to the tumor (Figure 1D). CT of the chest abdomen and pelvis with contrast was negative for malignancy. Due to the ICA location in the anterior and medial locations, transsphenoidal access would place the patient at higher risk for carotid injury. We elected to perform tissue sampling via a stereotactic MRI-guided temporal lobe trajectory.

Operative technique

**Figure 2:**
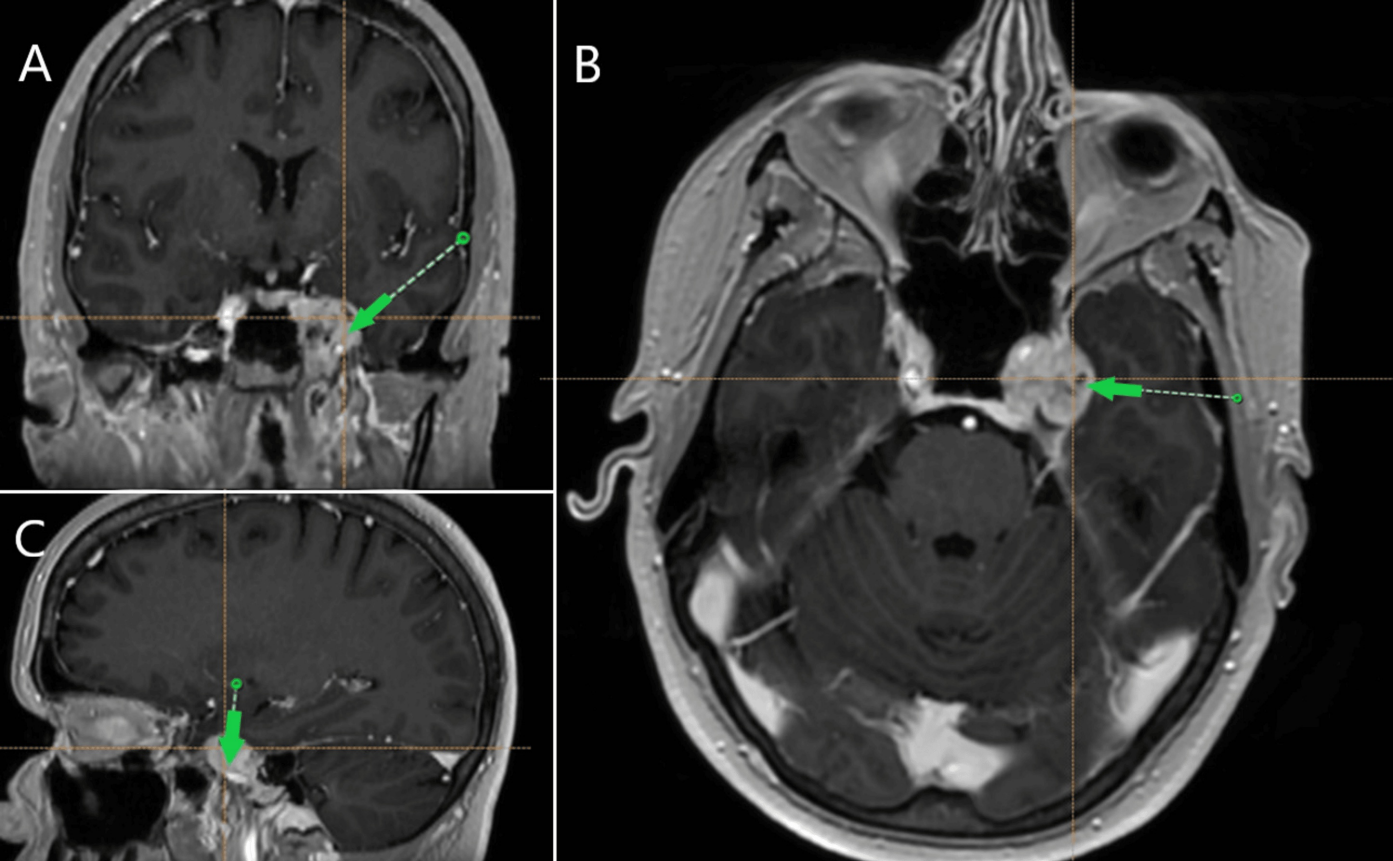
Trajectory planning Brain MRI via BrainLab® (Brainlab, Munich, Germany) with the target indicated by the green arrow at the lateral wall of the cavernous sinus on (A) coronal, (B) axial, and (C) sagittal views.

A stereotactic biopsy was planned for the patient. Informed consent was obtained prior to the surgery. Institutional review board evaluation was not needed for this case report per institutional protocol. Standard operating procedures were followed including preoperative stereotactic 3-dimensional T1-contrasted and fractional anisotropy/tractography MR sequences were obtained with fiducials in place. The patient was positioned supine with the head rotated to the left in a Mayfield clamp® (Integra LifeSciences Corporation, Princeton, USA) under general anesthesia. BrainLab® (Brainlab, Munich, Germany) was used for trajectory planning with entry through the superior temporal gyrus to the tumor-dural interface (Figure 2). A single burr hole was placed approximately 3.5 cm superior to the zygoma, immediately posterior to the pterion. A biopsy needle was introduced through the dura and superior temporal gyrus, but significant resistance was encountered when attempting to enter the region lateral and at the margin of the cavernous sinus.

**Figure 3:**
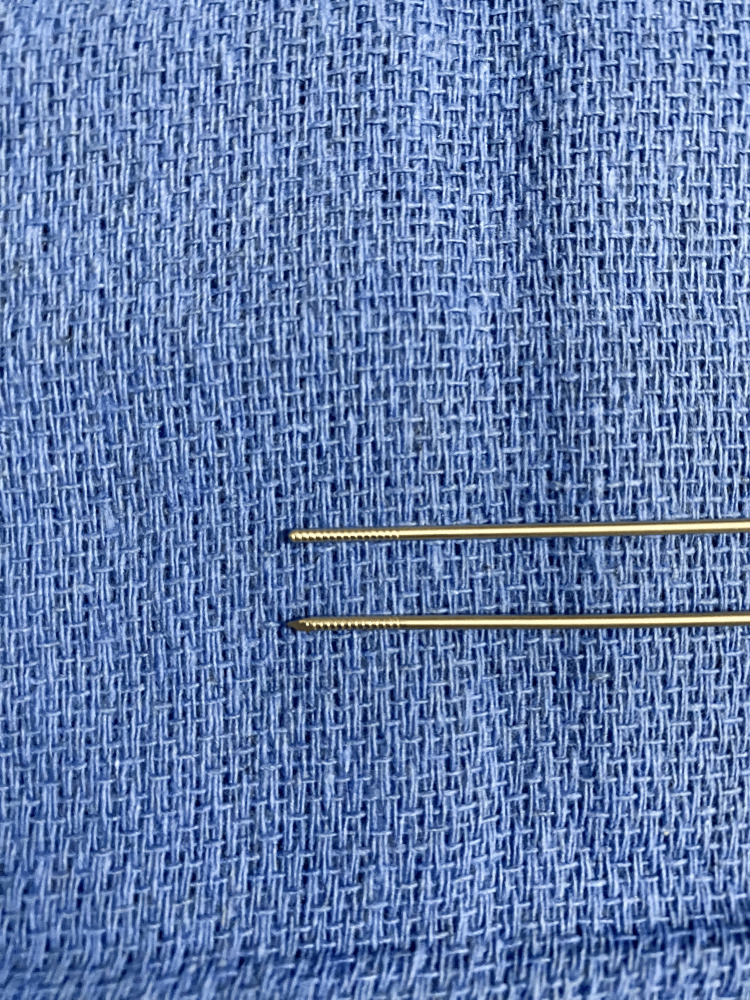
K-wire

A K-wire was used to penetrate the dura utilizing the same depth to target measurements. K-wires are stainless steel with a sharpened end typically used in spinal applications (Figure 3) Applying even pressure, a brief “give” is palpated. The K-wire is then withdrawn, and the biopsy needle is inserted along the same trajectory to the target assuming a breach of the lateral cavernous wall. No bleeding was observed with serial specimens obtained. The frozen section confirmed the appropriate target was sampled. The K-wire and biopsy needle were subsequently removed without hemorrhage. The site was irrigated copiously, and hemostatic agent/burr hole covers were placed. The incision was closed with 4-0 prolene.

Postoperative course

**Figure 4:**
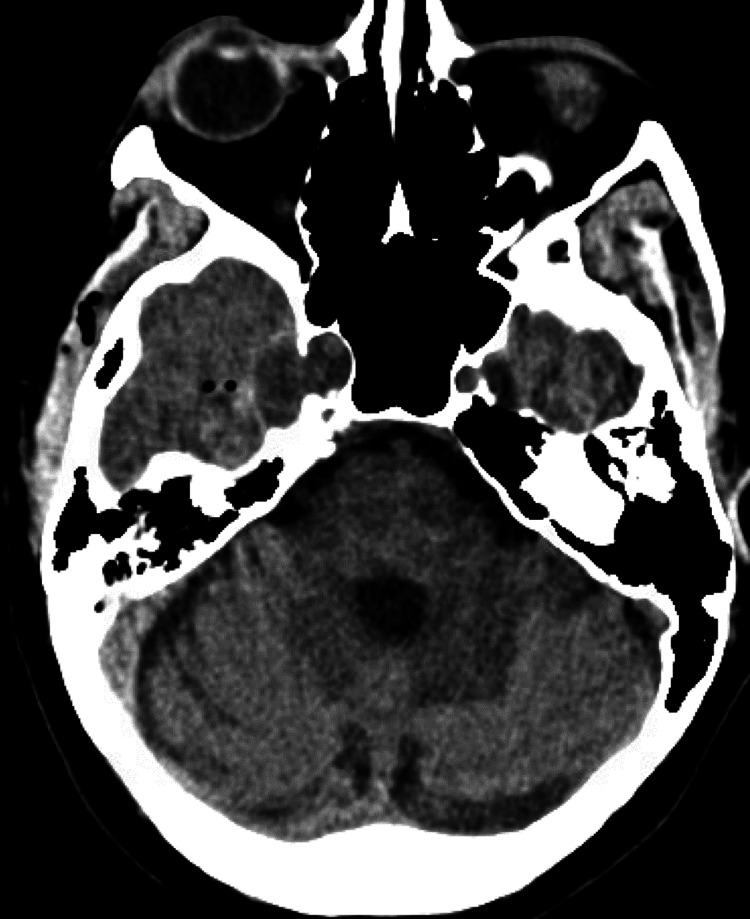
Postoperative imaging A non-contrast CT scan performed the morning following surgery.

Postoperatively, the patient did not endorse or exhibit any new symptoms and stable right abducens palsy. She was monitored overnight on the surgical step-down/intermediate unit. A non-contrast head CT (Figure 4) the following morning did not show any hemorrhage or stroke and she was discharged later that day. The final pathology was consistent with grade 2 chondrosarcoma. She denied any new symptoms and reported improved diplopia with prism glasses at one month follow-up. She started radiation therapy the following day - 30 fractions of external beam radiation at 180 cGy/fraction (total 4,140 cGgy) - and reports continued visual improvement.

## Discussion

The literature reports numerous petroclival approaches including (from anterior to posterior); endoscopic transsphenoidal, transoral, anterior percutaneous, middle fossa, and posterior fossa - traversing the cerebellar peduncle [1,2,3,5,6]. All approaches depend heavily on ICA location with respect to the tumor. Transsphenoidal and transoral approaches are preferred when pathology is located centrally, in the sella but require general anesthesia and risk cerebrospinal fluid (CSF) leak [7]. Most anterolateral percutaneous approaches begin beneath the zygomatic process, above the mandibular notch [8], and may be performed under local anesthetic [9]. This typically requires drilling the mastoid and greater sphenoid wing with a superior trajectory limited by the dural base/ICA position [3,9]. Finally, a posterior transcerebellar peduncle trajectory may be considered in cases of extension into the prepontine cistern, but proximity to the basilar artery, its branches, and cranial nerves limit frequent use.

Magrassi et al. detail the drawbacks of the traditional lateral/oblique approaches, citing long trajectories and increased ICA injury risk [3]. They present an intriguing case wherein their patient refused anesthesia, thus preventing the anterior approach. Instead, they entered 5 mm superior to the zygoma, at the middle fossa floor, and traversed just inferior to the lacerum segment of the ICA.

Our case had limited access inferiorly or anteriorly based on anatomy, but a favorable window adjacent to the medial temporal lobe. Therefore, an intra-axial intradural corridor provided a straightforward minimal-risk approach to obtaining the surgical specimen. The only prerequisite however is the sharp tip of the K-wire to penetrate the dura using this approach.

Our technique does have some theoretical risks: vertebral body breaches with K-wires are reported in the spine literature, albeit rarely [10,11], therefore surgeons must use minimal force to achieve safe results. K-wire fracture rates as high as 3% are reported in orthopedic literature [12]. However, these numbers reflect K-wires utilized for long-term stabilization, not intraparenchymal or intradural targets. Finally, K-wires theoretically increase the risk of intracranial hemorrhage, likely paralleling deep brain stimulation lead placement (1-10%, though most studies are on the lower end) [13,14]. These risks are arguably justified if K-wires permit safer trajectories by avoiding the ICA and critical neural structures.

## Conclusions

We report the first K-wire-assisted intra-axial intradural cavernous sinus biopsy confirming a grade II chondrosarcoma. The K-wire’s sharp end and small diameter allow safe access to intradural pathology and should be considered an adjunct to standard techniques when standard biopsy needles cannot penetrate dural boundaries.
